# Study on Microbial Community Succession and Functional Analysis during Biodegradation of Mushroom Residue

**DOI:** 10.1155/2021/6620574

**Published:** 2021-07-12

**Authors:** Chaonan Wang, Yuxin Wang, Hua Ru, Ting He, Nan Sun

**Affiliations:** College of Water Resources & Civil Engineering, China Agricultural University, Haidian, Beijing 100083, China

## Abstract

In this study, 16S rRNA high-throughput sequencing technology was used to analyze the composition and diversity of bacterial and fungal communities in mushroom residue samples at different composting stages. During the composting process, the maximum temperature in the center of the pile can reach 52.4°C, and the temperature above 50°C has been maintained for about 8 days. The results showed that *Actinobacteria*, *Firmicutes*, *Proteobacteria*, *Bacteroidetes*, and *Chloroflexi* were the main microorganisms in the composting process, accounting for 98.9%-99.7% of the total bacteria. Furthermore, in order to obtain the protein expressed in each stage of composting, the nonstandard quantitative method (label free) was used to analyze it quantitatively by mass spectrometry, anda total of 22815 proteins were identified. It indicated that the number of identified proteins related to cellulose decomposition and the number of differentially expressed proteins were significantly enriched, and the functional proteins related to cellulose decomposition had significant stage correspondence.

## 1. Introduction

China is the world's largest producer and consumer of edible fungi. In 2018, output of edible fungi in China had reached 37.12 million tons, accounting for more than 70% of the total global market [1]. The cultural materials used in the production of edible fungi include branches, sawdust, straw, corncob, rice husk, cotton husk, bran and so on. Mushroom residue is a mixture of mycelium and cultivation material left after the collection of edible fungi. In addition to abundant carbohydrate (cellulose, lignin, etc.) and nutrient products secreted by microbial metabolism, mushroom residue is also rich in bacterial protein, nitrogen, phosphorus, potassium, and other mineral elements, which is a valuable agricultural renewable resources [2]. Some studies have shown that mushroom residue is not only a by-product produced by the mushroom industry but also a composting resource to be developed as farmland organic fertilizer [3].

The essence of composting is solid-state fermentation, which is the process of transforming various complex organic matters such as cellulose and lignin into soluble nutrients and humus under the action of a variety of microorganisms. The compost degradation process is complex, and the types of microorganisms involved are diverse [4–6]. So, traditional methods cannot comprehensively and thoroughly study these habitat systems. The emergence of a new generation of high-throughput sequencing technology based on the Illumina platform can more comprehensively and accurately describe the microbial community information in decomposed compost and provide an effective means for exploring the composition and function of microbial community [7, 8]. Because of the complexity and diversity of microflora and functions in saprophytic environment, the specific expression and function of environmental microorganism genes under complex environmental conditions cannot be revealed effectively by metagenomics research [9, 10].

Metaproteomics is defined as the sum of all proteins of environmental microbiome in a specific time, and it is a new research direction in the field of environmental microbiology after macrogenomics [11–13]. Although functions can be characterized by genes and transcripts, the abundance measuring of proteins can more accurately and directly characterize the functional activities of cells or communities in a particular spacetime [14]. Proteomics techniques rely on shotgun quantification of mass and abundance of peptides by mass spectrometry. In short, the amino acid sequence and any posttranslational modification, such as phosphorylation, can be revealed by the fragmented form of the peptide and then compared with the reference database of sequence homology. Compared with nucleic acids, proteins reflect the actual functions of microbial metabolic responses and regulatory factors and give more direct and specific information about microbial activity than functional genes or even mRNA, which is a powerful supplement to functional genes [15–17]. In addition to the study of individual microorganisms, the application of proteomics technology to complex groups such as microbial communities is deepened [18, 19]. In the composting process, the microbial community will secrete different extracellular enzymes systematically. The analysis of these extracellular enzymes by metaproteomics sequencing technology can locate the corresponding microbial niche in the compost and then determine the role of these microorganisms in the composting process [20, 21]. In the research of natural corn stalk composting, the proteomics technology was used to find that pathogenic bacteria such as Acinetobacter and its abundance decreased significantly with the fermentation time [22]. In the study of municipal solid waste composting, it was found that the microbial community was undergoing dynamic changes throughout the composting process, among which the number of Bacillus, Actinomycetes, and Yeasts would increase significantly, and the key microorganisms that degrade cellulose at different composting stages are not the same [23, 24].

Since the expression of proteins is a reflection of specific microbial activities in a specific ecosystem, metaproteomics has greater potential in the process of microbial community function analysis [25–27]. This kind of research can link the composition of microbial communities with their functions. By studying the gene expression in different time and space, we can better understand the microbial community [28, 29]. Among the four research levels of genome, transcriptome, proteome, and metabolome, Macroproteomics is the most comprehensive and appropriate method to study the dynamic change of microbial community, and it is also the most important step to study the metabolic pathway and regulatory mechanism in metabonomics, which has great biological significance [30–32]. Nontargeted quantitative proteomics is a quantitative technique for the indiscriminate analysis of all proteins in a sample. Depending on whether the protein or peptide is labeled, nontargeted quantitative proteomics techniques can be divided into nonlabeled (label-free) and labeled (stable isotope labeling) quantitative techniques [33].

In this study, through 16S rRNA high-throughput sequencing technology, the bacterial and fungal communities in the samples were determined, the composition and distribution of microbial communities in different composting stages were studied, and the high-abundance microbial species in each composting stage were screened. At the same time, the protein types in compost were identified by label-free method, the protein expression differences in each stage were analyzed, the protein expression with cellulose decomposition function was identified, and the differential expression in different compost stages was described, and then, the molecular regulation mechanism of cellulose decomposition in each stage of compost was revealed from the protein level.

## 2. Materials and Methods

### 2.1. Introduction of Composting Equipment

Fresh mushroom residue (Flammulina Velutipes residue) was collected from the Beijing Hualu Biotechnology Company. And then, it was screened by 20-mesh sieves and stored as experimental composting material. Initial nutrient content of mushroom residue to be composted is shown in Table 1.

**Figure 1:**
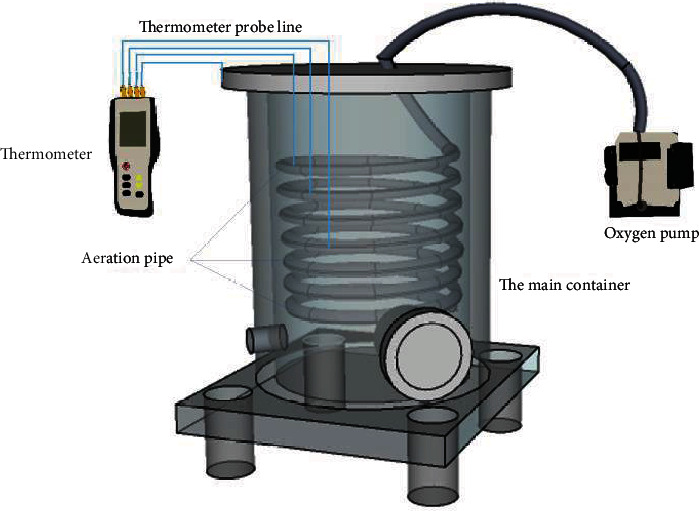
The schematics of aerobic composting equipment.

Aerobic composting equipment (China Patent No. 201720930414.3) was used in the experiment. The effective volume of the experimental device was about 80 L (in which the inner diameter was 210 mm and the height was 600 mm) (Figure 1).

Aerobic composting reactor is mainly composed of three parts: temperature monitoring system, ventilation system, and main reactor vessel. The composting experiment was conducted in 310 Laboratory, College of Water Conservancy and Civil Engineering, China Agricultural University. Each composting cycle lasted 40 days; pile-stirring and water-charging were carried out on the fifteenth and twenty-fifth days, respectively. According to the preliminary experiment, the ventilation rate of this study is set to 2 L/min and ventilating continuously for two hours every day, selected at 9:00-10:00 in the morning and 21:00-22:00 in the evening, respectively.

### 2.2. Sampling Method

#### 2.2.1. Sampling for Physicochemical Properties

According to the change of temperature, the composting process can be divided into four stages: warming stage, high temperature keeping stage, cooling stage, and final stage. Samples were taken at the top (10 cm below the surface), the middle (30 cm below the surface), and the bottom (50 cm below the surface) of the composting equipment in different periods. The specific sampling time is the 1st, 5th, 10th, 15th, 20th, 25th, 30th, 35th, and 40th days. Samples are packed in sampling bags, sealed, and stored in a refrigerator at -20°C for subsequent analysis and determination experiments.

#### 2.2.2. Sampling for Metaproteomics, 16s, and Its Sequencing

During mushroom residue composting, according to the indication of temperature, sampling was arranged on the 3rd day (warming stage), the 12th day (high temperature keeping stage), the 23rd day (cooling stage) and the 40th day (final stage) of composting. Sample numbers of bacteria and fungi were set up as D3B, D12B, D23B, and D40B and D3F, D12F, D23F, and D40F, respectively.

### 2.3. Test Methods for Physicochemical Properties

#### 2.3.1. Temperature

The four-channel contact thermometer was embedded in the composting material in advance, and the ambient temperature around the composting equipment was measured at the same time. The temperature changes were recorded at 9:00 am and 9:00 pm in each day during the composting period.

#### 2.3.2. Moisture Content

Put about 5 g fresh sample into the crucible, weigh its mass, then put it in 105°C electric heating blast dryer to dry to constant mass, measure the mass of samples after drying, and calculate the moisture content(W)according to Equation (1).

(1) $$\begin{array}{c} W\left(\%\right)=\frac{A_{0}-A}{A_{0}-C}\times 100, \end{array}$$

where

*A*_0_: the mass of crucible and sample before drying (g)

*A*: the mass of crucible and sample after drying (g)

*C*: crucible mass before drying (g).

#### 2.3.3. pH Value

2 g of air-dried sample was weighed and extracted at the ratio of 1 : 20 of sample and distilled water. After 30 minutes, the pH was determined with pH meter.

#### 2.3.4. *C*/*N* Ratio

After grinding the air-dried compost sample and passing through 80-mesh sieves, the content of total carbon and total nitrogen in the compost sample was analyzed.

#### 2.3.5. GI Index

20 g fresh compost samples were weighed and extracted at the ratio of 1 : 10 of sample and distilled water to obtain compost extract. Using distilled water as control, cucumber seeds (Variety Zhongnong 20, from Zhongshu Seed Technology Co., Ltd.) were cultured in compost extract. After 48 hours, the germination rate and root length of cucumber seeds were measured. GI index was calculated according to Equation (2).

(2) $$\begin{array}{c} \text{GI}\left(\%\right)=\frac{F_{0}\times E}{F\times E}\times 100, \end{array}$$

where

*F*_0_: germination rate in the extract (%)

*F*: germination rate in the distilled water (%)

*E*: seed root length (mm).

### 2.4. High-Throughput Sequencing

#### 2.4.1. DNA Extraction

Total DNA was extracted by E.Z.N.A. & soil kit, DNA concentration and purity were detected by nano-Drop 2000. Total DNA was detected by 1% agarose gel electrophoresis and stored at -80°C for subsequent experiments.

#### 2.4.2. PCR Amplification

The accuracy and reliability of sequencing data can be ensured by reducing the number of cycles as much as possible. 27 cycles were selected in this experiment. The main reaction parameters were 95°C, 3 min; 95°C, 30 s; 55°C, 30 s; 72°C, 45 s; and 72°C, 10 min. The amplified primers selected for bacteria are 338F:5′-ACTCCTACGGGAGGCAGCAG-3′ and 806R: 5′-GGACTACHVGGGTWTCTAAT-3′. The amplified primers for fungi are ITS1F: 5′-CTTGGTCATTTAGAGGA AGTAA-3′ and ITS2R: 5′-GCTGC GTTCTTCATCGATGC-3′.

#### 2.4.3. Illumina Sequencing

Amplifiers were isolated by 2% agarose gel. The product was purified in AxyPrep DNA Gel Extraction Kit and eluted with Tris-HCl. The amplification effect of PCR was examined by 2% agarose electrophoresis. Finally, the amplifiers were quantified in QuantiFluor ST.

#### 2.4.4. Amplified Sequence Extraction and OTU Clustering

The original amplifier sequencing data was uploaded to the NCBI Sequence Read Archive (SRA) database (login account: PRJNA544983). The original Fastq file is reoptimized, filtered by Trimmomatic, and merged by FLASH. Sequencing data were clustered into operational taxa (OTU) in UPARSE (version 7.1 http://drive5.com/uparse/) according to the 97% similarity cut-off value. UCHIME is used to identify and prune chimeric sequences. Bacterial sequencing data were classified by RDP classifier algorithm (http://rdp.cme.msu.edu/) corresponding to Silva (version 128) 16S rRNA database to analyze the classification of each 16S rRNA gene sequence under 70% confidence threshold. Corresponding to Unite (Version 7.0) database, fungal sequencing data were classified using RDP classifier algorithm (http://rdp.cme.msu.edu/) under 70% confidence threshold.

### 2.5. Protein Extraction and Mass Spectrometry

1. A suitable amount of compost sample was taken out under the freezing condition, transferred to MixPlus oscillator tube, and added appropriate amount of BPP solution. The compost sample was ground into fine powder by low temperature grinder.
2. 1 g sample was added into centrifugal tube, 3 mL extracting buffer was added, and the supernatant was shaken in an ice bath for 20 minutes. The supernatant was shaken in ice bath for 10 minutes with equal volume of protein denaturant (Tris-saturated phenol). The sample was centrifuged at 12 000 rpm and 4°C for 20 minutes. The top phenol layer was transferred to a new centrifugal tube, and attention was paid to avoid touching the middle layer. In this new centrifugal tube, equal volume BPP solution was added to shake in ice bath for 10 minutes. The sample was centrifuged at 12 000 rpm and 4°C for 20 minutes, and then, the phenolic phase of the upper layer was transferred to another new centrifugal tube. In this new centrifuge tube, 5 times of equal volume of precipitation solution is added to fully oscillate the centrifuge tube and precipitate for a whole night at -20°C. The samples were centrifuged at 12000 rpm and 4°C for 20 minutes to precipitate proteins. At the same time, the precipitates were rinsed twice with precooled acetone. Finally, these precipitates are dried by freeze-vacuum dryer.
3. Protein lysate was used to dissolve protein precipitation and then centrifuged at 12000 rpm and 4°C to obtain the supernatant of total protein.

In this experiment, BCA (bicinchoninic acid) method was used to quantify the protein in the samples. Bovine serum albumin (BSA) with different concentrations was prepared according to the standard protein provided by Thermo Scientific Pierce BCA kit. The SPECTRA MAX analyzer was used to read out the absorbance of a standard sample solution at 562 nm to make standard curves, and then, the test of the experimental samples was carried out. Meanwhile, the total protease obtained from the above sample was digested into peptide segments and detected by Thermo Scientific Q-extractive tandem mass spectrometer.

### 2.6. Database Searching

The original mass spectrometry data (raw data) was submitted to the Proteome Discoverer server (Proteome Discoverer TM Software 2.2), and the dominant species obtained from diversity detection were incorporated into the database, and then, the data were searched and compared through the database.

### 2.7. Annotation of Protein Function

The proteins that need to be identified are compared with the GO (Gene Ontology, http://www.geneontology.org) comprehensive database, and the functional annotation analysis of all identified differential proteins were conducted. COG (Cluster of Orthologous Groups of proteins, https://www.ncbi.nlm.nih.gov/COG/) online database is used for direct homology classification of proteins, and differential proteins mainly involved in cellulose degradation are screened out.

## 3. Results

### 3.1. Physicochemical Properties of the Compost Samples

**Figure 2:**
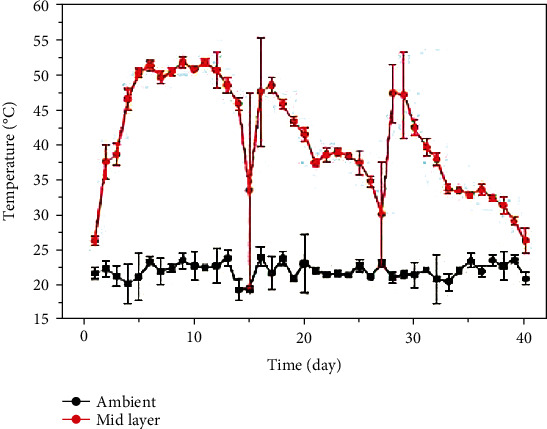
Temperature variation during composting of mushroom residue.

During the biodegradation process of mushroom residue, the initial temperature of the compost material (0-5 days) rose rapidly and reached 50°C on the fifth day. The highest temperature in the composting cycle was 52.4°C, and the material temperature in the compost pile remained above 50°C for 8 days, which indicated effective biodegradation effect and microbial activity. In addition, pile-stirring and water-charging were conducted on the 15th and 27th days, so the temperature of materials in the composting equipment decreased significantly on the 15th and 27th days (Figure 2).

**Figure 3:**
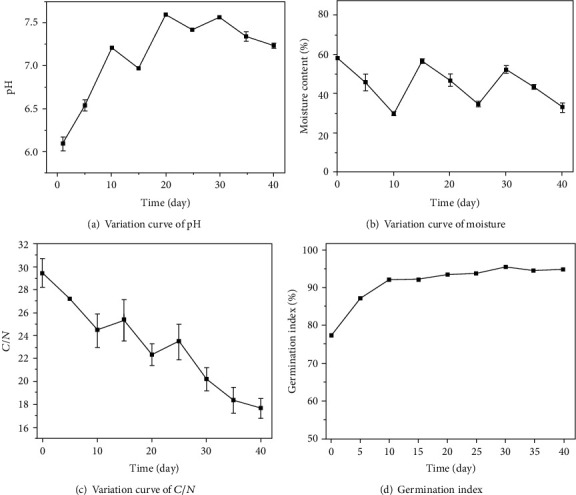
Changes of physicochemical properties of mushroom residue during composting.

The biodegradation process can be divided into four stages: 1-4 days for the warming stage, 5-12 days for the high temperature keeping stage, 13-29 days for the cooling stage, *and* 30-40 days for the final stage. With the increase of composting time, the pH of composting materials increased first and then decreased, showing the characteristics of changing from weak acidity to weak alkalinity (Figure 3(a)). Water in the biodegradation process can regulate the inner temperature of the composting pile (Figure 3(b)). With prolonging of degradation period, it was necessary to supplement water in the composting pile in order to maintain the normal growth and metabolism of microorganisms. In this experiment, the initial *C*/*N* ratio of compost material was adjusted to 29.47, and the C/N ratio decreased to 17.66 at the end of composting (Figure 3(c)). GI value increased gradually with the increase of composting days (Figure 3(d)), and finally, GI value increased from 77.34% to 95%.

On the 15th and 27th days of the composting process, mushroom residue was fully stirred, mixed, and watered in order to degrade the raw materials as quickly as possible. Temperature, pH, moisture, *C*/*N* ratio, and GI index of composting materials varied with the mixing and water increasing process. After stirring over the pile, the temperature rose sharply, which might be related to the fact that the raw materials close to the side wall of the composting equipment were mixed into the central part, resulting in the strong metabolic activity of the microorganisms.

### 3.2. General Characteristics of the Pyrosequencing

**Figure 4:**
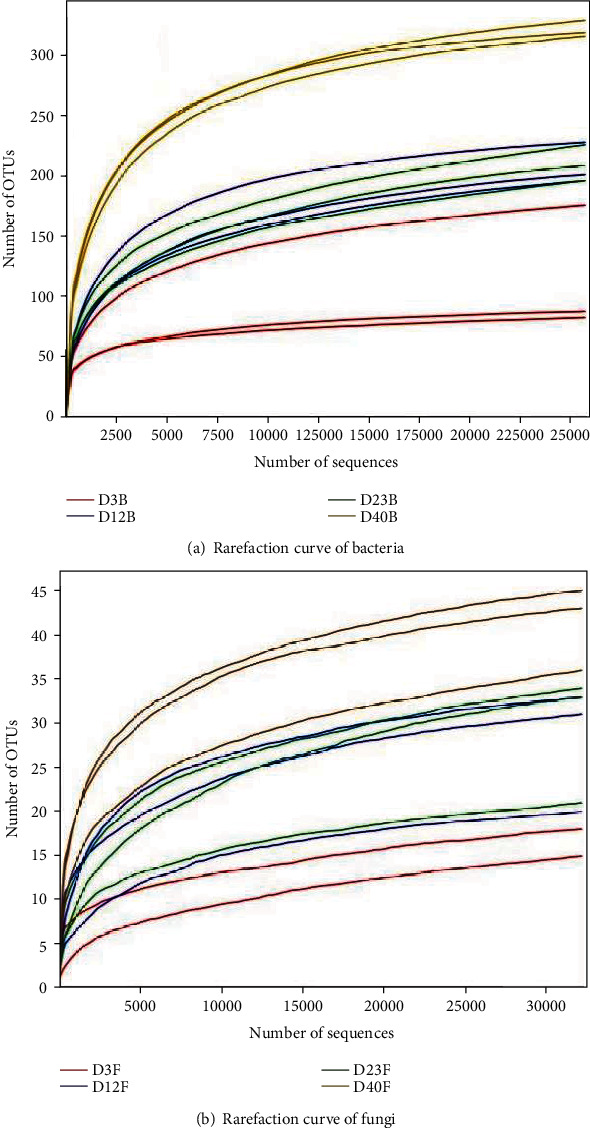
Rarefaction curve of microbial community.

The bacteria rarefaction curve (Figure 4(a)) tended to be flat with the increase of sequencing data, which indicated that the gene sequences obtained from each composting sample were reasonable. The existing sequencing quantity covered most OTUs in compost samples. The fungal rarefaction curve (Figure 4(b)) tended to be less gentle than that of bacteria with the increase of sequencing quantity. At the same time, the dilution curve also showed that the number of sequencing was sufficient, and most microbial species could be detected, which could reflect the changes of microbial community structure in the composting process. Before calculating alpha diversity, the sample data were normalized in order to control the difference of sequences quantity in the composting samples.

The optimum sequences, OTU numbers, and diversity index of different stages in the composting process were shown in Table 2. Through sequencing, 450888 bacterial sequences and 543669 fungal sequences were obtained in this experiment. Using 97% similarity as threshold, 416 different bacterial OTUs and 79 different fungal OTUs were obtained. The numbers of bacterial OTUs in different stages of biodegradation were about 100-300, and the numbers of fungal OTUs in different stages of biodegradation were about 20-50.

**Figure 5:**
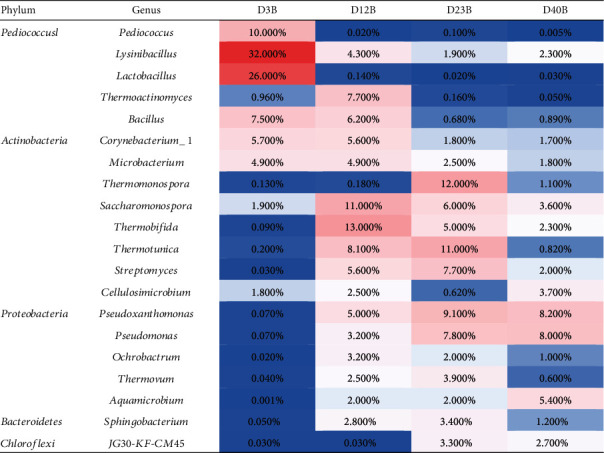
Succession of dominant bacteria in different stages of composting. In this figure, a certain color gradient is used to represent the proportion of species, red represents high-abundance species, and blue represents lower abundance.

In order to further understand the richness and diversity of microorganisms in the composting process, it is necessary to calculate Chao1 value and Shannon index at different stages. The Chao1 value is proportional to the microbial community richness. From Figure 5, it can be seen that the Chao1 value of bacteria and fungi increased by prolonging of composting time. The Shannon index on the third day of composting was the lowest. In contrast, Shannon index on the 40th day of composting was the highest.

### 3.3. Composition of Bacterial Community

**Figure 6:**
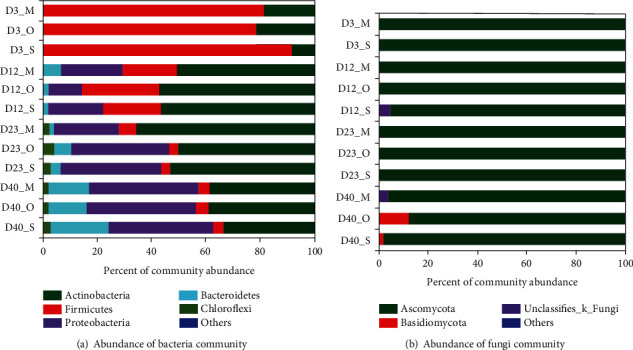
Microbial community structure.

It showed that there are 15 bacterial phylums in 12 compost samples, among which the top five are *Actinobacteria*, *Firmicutes*, *Proteobacteria*, *Bacteroidetes*, and *Chloroflexi*, which account for 98.9% to 99.7% of the total bacteria (Figure 6(a)). *Actinomycetes* accounted for a relatively small proportion in the warming stage, and whose richness increased to the top 1 at the level of phylum in the high temperature keeping stage. The richness of *Firmicutes* showed a sharp downward trend with prolonging of composting time. The richness of *Proteobacteria* and *Bacteroidetes* continued to increase with compost going on, and *Proteobacteria* became the largest phylum at the final stage. The richness of *Chloroflexi* in the cooling and final stages was higher than that of warming stage. As shown in Figure 6(b), four fungal phylum were detected in the samples, of which *Ascomycota* and *Basidiomycota* had high richnesses, which reached about 98.3%-99.5%. *Ascomycetes* dominated the whole composting process, and *Basidiomycetes* increased gradually with the continuing of compost.

The communities whose relative richnesses over 1% were shown in the Figure 6. The intermediate rank of taxonomic pedigrees in taxonomic databases without scientific names is marked by norank.

During the composting process, the relative richnesses of bacteria at the generic level was shown in Figure 5. At the initial stage of composting, the top 6 abundant genera were *Lysinibacillus*, *Lactobacillus*, *Bacillus*, *Corynebacterium*, *Pediococcus*, and *Mirobacterium*. In the high temperature keeping stage, the relatively abundant bacteria were *Thermobifida*, *Thermotunica*, *Thermoactinomyces*, *Saccharomonospora*, *Bacillus*, and *Streptomyces*. *Pseudoxanthomonas*, *Thermomonospora*, *Pseudomonas*, *Thermotunica*, *Streptomyces*, and *Saccharomonospora* were the genera with higher abundance at the cooling stage. At the final stage, *Pseudoxanthomonas*, *Aquamicrobium*, *Ruminofilibacter*, *Pseudomonas*, *Nonomuraea*, and *Cellulosimicrobium* were the dominant genera.

At the generic level, the richnesses of dominant fungi in different stages of composting were shown in Table 3. *Thermomyces* was the most abundant genus at the high temperature keeping stage, cooling stage, and final stage. *Unclassified_o_Saccharomycetales* was the most abundant at the warming stage, but whose richness decreased sharply at the high temperature keeping stage. The richness of *Aspergillus* rose with the continuing of compost and reached 30% at the final stage. The richness of *unclassified_o_Sordariales* was higher at the high temperature keeping stage, and reached 14.4% .

### 3.4. Identification and Analysis of Metaproteomics

**Figure 7:**
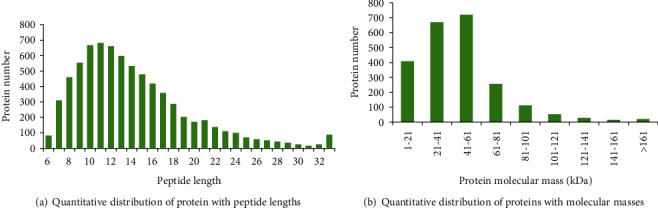
Peptide quality control information.

From the perspective of peptide control information, it indicated that most of proteins identified in this experiment contained no more than 11 peptide segments (about 90%). The quantitative distribution of protein with peptide lengths was shown in Figure 7(a)), and the number of proteins containing 8-16 peptide amino acid residues reached 5050.

In this experiment, totally, 51034 tandem mass (MA/MS) spectra of protein were detected. Meanwhile, a total of 7405 peptide segments, 22815 proteins, and 2281 proteomes were identified by NCBI database and Masot software. As shown in Figure 7(b), the molecular masses of differential proteins were mainly concentrated in the range of 1-81 kDa, and totally, about 2053 differential proteins were detected. Among them, there were 719 proteins with relative molecular mass range from 41 to 61 kDa.

According to fold change of protein expression >1.200 or <0.833 and *P* value < 0.05, 2212 differentially expressed proteins were screened. It indicated that there were significant differences in protein expression among different biodegradation stages. There were 2117 differentially expressed proteins at the warming stage compared with that of high temperature keeping stage (Table 4); Among them, 1181 belonged to upregulated proteins (including 300 specifically upregulated proteins) and 936 belonged to downregulated proteins (including 419 specifically downregulated proteins). There were 1853 differentially expressed proteins at the high temperature keeping stage compared with that of cooling stage. Among them, 1209 belonged to upregulated proteins (including 473 specifically upregulated proteins) and 644 belonged to downregulated proteins (including 138 specifically downregulated proteins). There were 1886 differentially expressed proteins at the cooling stage compared with that of final stage. Among them, 888 belonged to upregulated proteins (including 156 specifically upregulated proteins) and 998 belonged to downregulated proteins (including 490 specifically downregulated proteins).

**Figure 8:**
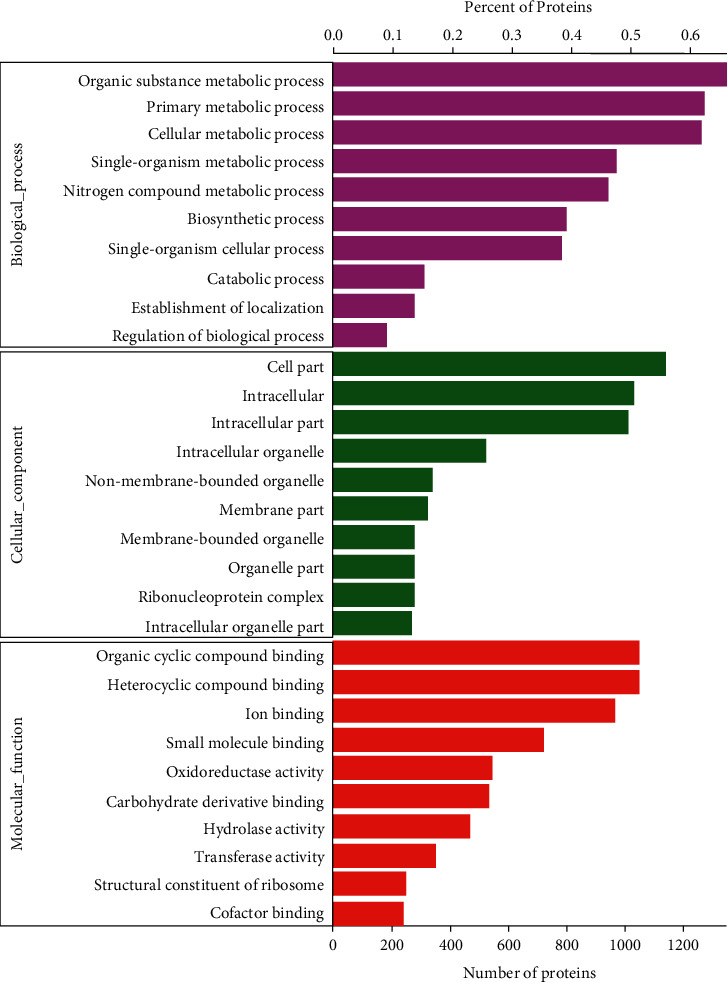
GO classification of identified proteins.

Total GO function annotations of the proteins identified at every degradation stage are shown in Figure 8. It was obviously that the main functions of protein enriching were organic substance metabolic process, primary metabolic process, and cellular metabolic process in the biological process. In molecular function, the identified proteins are mainly concentrated in organic cyclic compound binding, heterocyclic compound binding, and ion binding.

**Figure 9:**
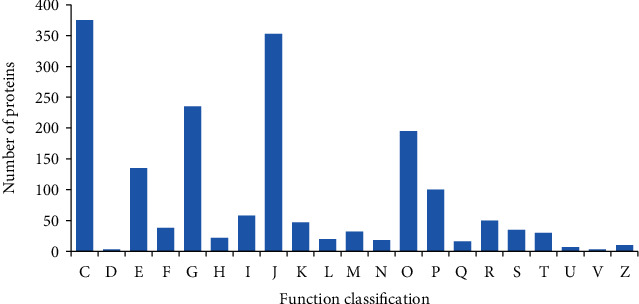
COG coverage of identified proteins. The abscissa represents the functional classification of COG (expressed in capital letter A-Z), and the ordinate represents the number of proteins with such functions. Among them, A: RNA processing and modification; B: chromatin structure and dynamics; C: energy production and conversion; D: cell cycle control, cell division, chromosome partitioning; E: amino acid transport and metabolism; F: nucleotide transport and metabolism; G: Carbohydrate transport and metabolism; H: coenzyme transport and metabolism; I: lipid transport and metabolism; J: translation, ribosomal structure and biogenesis; K: transcription; L: replication, recombination and repair; M: cell wall/membrane/envelope biogenesis; N: cell motility; O: posttranslational modification, protein turnover, chaperones; P: inorganic ion transport and metabolism; Q: secondary metabolites biosynthesis, transport and catabolism; R: general function prediction only; S: function unknown; T: signal transduction mechanisms; U: intracellular trafficking, secretion, and vesicular transport; V: defence mechanisms; W: extracellular structures; Y: nuclear strctures; Z: cytoskeleton.

As shown in Figure 9, the results of COG homologous functional classification showed that most of the functions of orthologous proteins identified during composting were concentrated in energy production and conversion (C, 21.29%), translation, ribosomal structure and biogenesis (J, 20.32%), carbohydrate transport and metabolism (G, 13.22%), and posttranslation modification, protein turnover, and molecular chaperones (O, 11.22%).

## 4. Discussion

**Figure 10:**
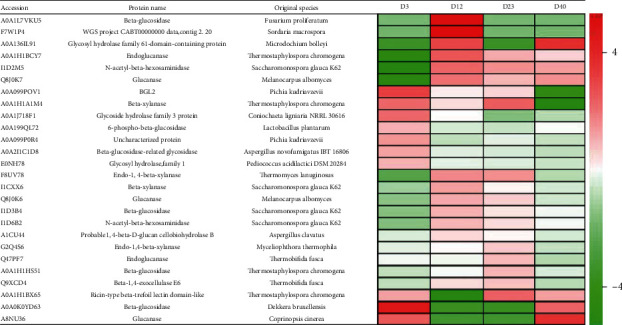
Differential protein of cellulose decomposition at various stages of composting.

The proteomic analysis of composting indicated that the number of identified proteins and differentially expressed proteins related to cellulose decomposition were significantly enriched, which played an important role in the degradation of cellulose and other organic matter in mushroom residue (Figure 10). The functional proteins related to cellulose decomposition at different stages have significant periodic correlation. At the warming stage, the expressions of *BGL2*, *Beta-xylanase*, *Glycosidehydrolase family 3 protein*, *Beta-glucosidase*, and *Glucanase* were significantly higher than those at the high temperature keeping stage. It revealed that with the increase of temperature, the activity of some microorganisms decreases gradually, while that of other microorganisms adapted to high temperature increases gradually, so the expressions of proteins related to cellulose decomposition increased.

At the high temperature keeping stage, the expressions of *Beta-glucosidase*, *Glycosylhydrolase family 61-domain-containing protein*, *Endoglucanase* and *N-acetyl-beta-hexosaminidase* secreted by *Fusarium Proliferatum* increased significantly. However, the expressions of *β-glucosidase* and *Glucanase* secreted by *Ricin-typebeta-trefoil lectin domain-like* and *Dekkera bruxellensis* was significantly down regulated compared with that in the warming stage.

The expressions of *Beta-xylanase*, *Endo-1, 4-beta-xylanase*, and *Ricin-type beta-trefoil lectin domain-like* secreted by *Thermostaphylospora Chromogena* increased significantly during the cooling stage. However, the expressions of *Beta-xylanase*, *Glycosyl hydrolase family 61-domain-containing protein* secreted by *Fusarium Proliferatum* and *Beta-glucosidase* secreted by *Dekkera Bruxellensis* decreased significantly.

During the final stage, the expressions of *Glycosyl hydrolase family 61-domain-containing protein*, *Glucanase* secreted by *Coprinopsis Cinerea, Beta-glucosidase* secreted by *Dekkera Bruxellensis*, *N-acetyl-beta-hexosaminidase*, and other proteins increased greatly.

However, the expressions of *BGL2*, *Beta-xylanase* secreted by *Coprinopsis Cinerea*, *Beta-xylanase* secreted by *Fusarium proliferatum*, and other proteins decreased significantly. Although some proteases secreted by different microorganisms having the same function, their activities varied greatly among different stages. For example, the expression of beta-glucosidase secreted by *Fusarium proliferatum*was higher significantly at high temperature keeping stage, but lower at the other composting stages. However, the expression of Beta-glucosidase secreted by *Dekkera bruxellensis* was higher in the warming and final stages, but lower in the cooling stages.

## 5. Conclusions

During the composting process, the color of mushroom residue changed from yellow brown to dark gray and finally to dark black, and the whole texture changed from hard to fragile. During the composting process, the maximum temperature in the center of the pile can reach 52.4°C, and the temperature above 50°C has been maintained for about 8 days. The pH in the pile gradually changes from weak acid to weak alkaline. At the end of composting, the *C*/*N* ratio of compost materials decreased from 29.47 to 17.66.

With the increase of composting time, the number of OTUs detected gradually increased. *Actinobacteria*, *Firmicutes*, *Proteobacteria*, *Bacteroidetes*, and *Chloroflexi* were the main microorganisms in the composting process, accounting for 98.9%-99.7% of the total bacteria; *Ascomycota* and *Basidiomycota* were the most abundant fungi, accounting for 98.3% of the total fungi.

In order to obtain the proteins expressed differentially in each stage of composting, the nonstandard quantitative method (label free) was used to analyze it quantitatively by mass spectrometry. A total of 22815 proteins were identified and in which 2212 differential proteins were obtained. Furthermore, at the same time, the existence of some specific expression proteins also indicated that the responses of microorganisms in these composting stages were quite different.

## Figures and Tables

**Table 1:** Initial physical and chemical properties of composting materials.

	pH	Moisture content (%)	Organic matter content (%)	Total carbon content (%)	Total nitrogen content (%)	*C*/*N*
Mushroom residue	6.09	59.7	85.15	44.8	1.22	36.72

**Table 2:** Microbial OTU number and diversity index during composting.

Items		D3	D12	D23	D40
Sequences	Bacteria	37802 ± 6602.61	36493 ± 3071.4	38224.67 ± 586.82	37776.33 ± 2046.57
	Fungus	72641.5 ± 2180.01	45134.33 ± 1887.14	46487 ± 2378.53	41174 ± 7670.71
OTU number	Bacteria	118.33 ± 56.92	213.33 ± 21.94	216.33 ± 15.95	324 ± 6.24
	Fungus	22 ± 2.83	29.67 ± 6.66	31 ± 5.2	44 ± 5.57
Chao1	Bacteria	140.89 ± 65.54	234.21 ± 19.63	268.71 ± 37.83	347.12 ± 16.71
	Fungus	29 ± 2.83	31.53 ± 6.12	37.62 ± 2.05	50 ± 2
Shannon	Bacteria	2.73 ± 0.13	3.35 ± 0.2	3.43 ± 0.14	4.18 ± 0.09
	Fungus	0.25 ± 0.3	0.51 ± 0.19	0.66 ± 0.29	1.26 ± 0.19

**Table 3:** Succession of dominant fungi in different stages of composting.

Genus	Percentage (%)			
	D3F	D12F	D23F	D40F
*Thermomyces*	0.01	88.47	80.26	53.61
*Unclassified_o__Saccharomycetales*	95.02	0.07	0.01	0.01
*Aspergillus*	0.01	5.05	2.63	30.42
*Unclassified_o__Sordariales*	0.01	4.09	14.4	0.01
*Cephaliophora*	0.01	0.11	0.01	6.25

**Table 4:** Differential proteins in different stages of composting.

	Proteins statistics	D3 vs. D12	D12 vs. D23	D23 vs. D40
Upregulated proteins	Specifically upregulated proteins	300	473	156
	Coupregulated proteins	881	736	732
Downregulated proteins	Specifically downregulated proteins	419	138	490
	Codownregulated proteins	517	506	508
Total		2117	1853	1886

## Data Availability

Most data have been presented in the manuscript. Additional details or data that support the findings of this study are available from the corresponding author upon reasonable request.
